# Soft bioelectronics for wireless drug delivery: programmable, minimally invasive and closed-loop therapeutics

**DOI:** 10.1038/s44431-026-00041-w

**Published:** 2026-09-18

**Authors:** Feng Zhang, Fuchang Sun, Zheng Yan

**Affiliations:** 1 Department of Chemical and Biomedical Engineering, University of Missouri, Columbia, MO USA; 2 NextGen Precision Health, University of Missouri, Columbia, MO USA

**Keywords:** Biological techniques, Biotechnology, Engineering, Materials science, Nanoscience and technology

## Abstract

Soft bioelectronics is reshaping drug delivery from passive dosing to programmable, tissue-interfaced therapeutics. This Review examines the co-design of soft materials, wireless power, drug reservoirs, release mechanisms and feedback control for wearable and minimally invasive implantable therapeutic systems. We organize the field around key constraints including controllability, reversibility, payload capacity, dosing precision, drug stability and feedback latency, guiding the development of adaptive closed-loop drug delivery platforms for clinical translation.

## Introduction

Conventional pharmacotherapy still depends largely on oral medicines, injections, transdermal patches and implanted depots^1–3^. These routes have transformed clinical care, but most administer drugs according to schedules that only indirectly reflect the moment-to-moment state of disease^4,5^. Oral dosing is shaped by absorption, distribution and metabolism, resulting in plasma peaks, troughs and interpatient variability^6–8^. Injections can improve bioavailability but introduce pain, training requirements and adherence barriers. Implanted depots extend release but are often difficult to adjust after placement. The central limitation is therefore not simply delivery efficiency, but the inability to coordinate dose, timing and location with changing physiology.

Remote-triggered formulations, implantable reservoirs and wireless microdevices have shown that pharmacokinetics can be programmed rather than accepted as a fixed consequence of formulation chemistry^9,10^. However, programmable release creates a drug-delivery-specific control problem. Once a chemical payload is released, its local concentration, tissue distribution, clearance and off-target effects cannot usually be reversed as rapidly as an electrical stimulus can be switched off. A clinically useful system must therefore coordinate sensing, actuation and reservoir design with pharmacokinetic and pharmacodynamic latency.

**Fig. 1:**
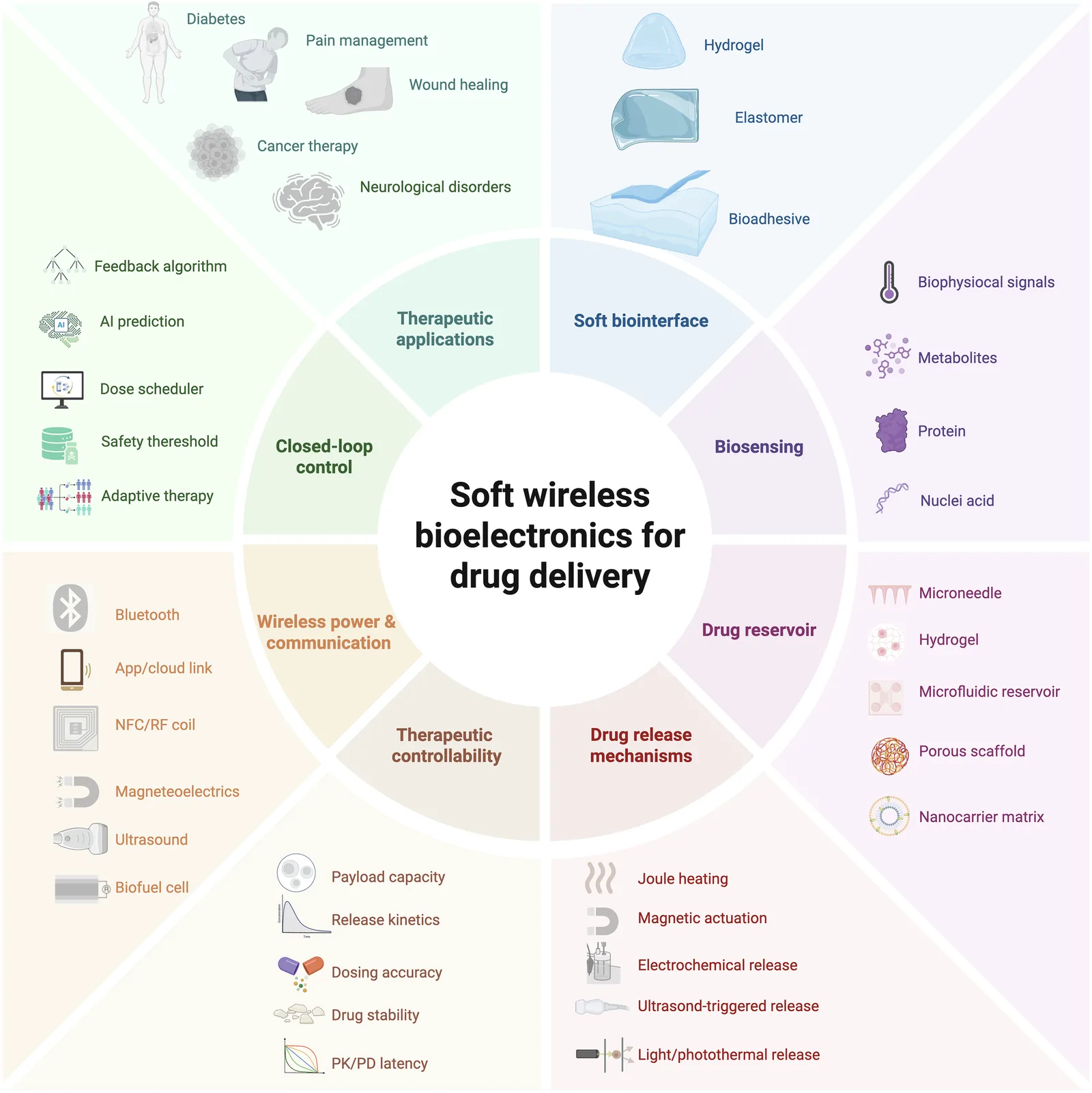
Conceptual framework for soft wireless bioelectronics in drug delivery. Soft wireless bioelectronic drug-delivery systems are integrated therapeutic loops rather than isolated release devices. The framework links soft bio-interfaces, biosensing, drug reservoirs, drug-release mechanisms, therapeutic controllability, wireless power and communication, closed-loop control and therapeutic applications.

Soft wireless bioelectronics offers a systems-level architecture for this transition from passive administration to adaptive, programmable therapy (Fig. 1). Soft biointerfaces, including hydrogels, elastomers, ultrathin polymers and bioadhesives, can mitigate mechanical mismatch and preserve contact with dynamic tissues^11–15^. Biosensing modules convert biophysical or biochemical signals into actionable inputs, whereas reservoirs, pumps, gates and microneedles translate electronic commands into chemical doses. Wireless power and communication decouple the therapeutic interface from external hardware. Closed-loop algorithms then link sensing to actuation, but their safety depends on the stability, capacity and reversibility of the drug-release module^16–18^.

This Review is positioned relative to recent progress of smart closed-loop drug delivery, wirelessly controlled delivery systems and intelligent miniaturized drug-delivery devices .Rather than re-cataloguing the full field, we ask how soft bioelectronic drug-delivery systems should be designed when tissue coupling, wireless operation and chemical dosing are inseparable. We first discuss soft and bioactive materials for stable device-tissue bio-interfaces. We then connect wireless power and communication to triggered release mechanisms, emphasizing a controllability and reversibility spectrum that spans one-shot gates, titratable pumps and refillable reservoirs. Finally, we examine wearable and minimally invasive platforms through the lens of clinical failure modes, including interface instability, payload exhaustion, dosing inaccuracy, feedback delay and regulatory complexity.

## Soft and bioactive materials for device-tissue interfaces

### Overcoming mechanical mismatch

**Fig. 2:**
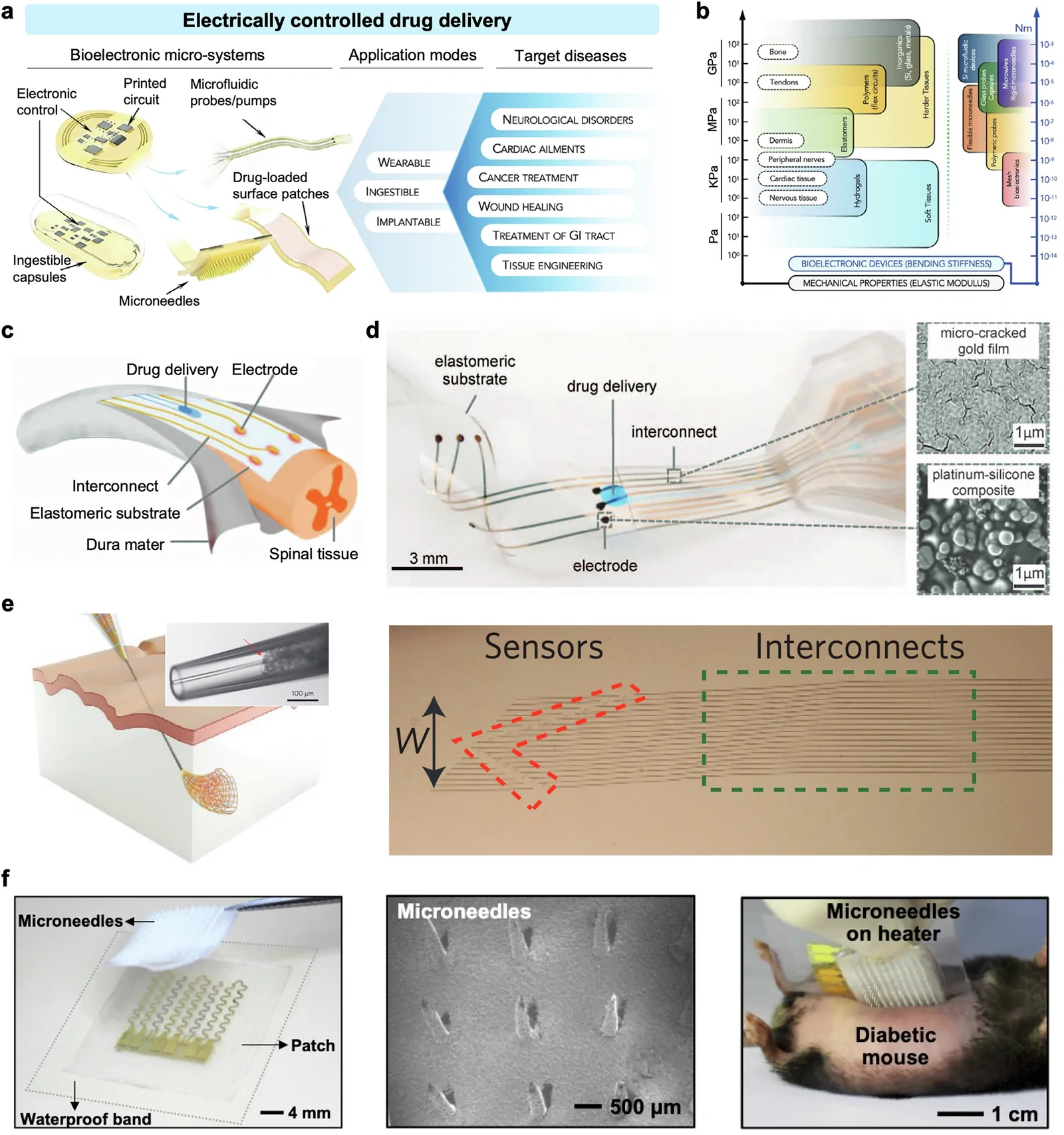
Soft and flexible bioelectronic platforms for electronically controlled drug delivery. **a** Schematic overview of electronically controlled drug-delivery micro-systems, including ingestible capsules, microfluidic pumps, drug-loaded surface patches and microneedle-based platforms, together with wearable, ingestible and implantable application modes. Reproduced with permission from ref. ^14^. Copyright 2023, Wiley-VCH GmbH. **b** Mechanical comparison between biological tissues and bioelectronic devices, highlighting the need to reduce bending stiffness and elastic-modulus mismatch for chronic tissue interfaces. Reproduced with permission from ref. ^14^. Copyright 2023, Wiley-VCH GmbH. **c**, **d** Electronic dura mater (e-dura) as a compliant neural interface that integrates stretchable interconnects, electrodes, and a microfluidic drug-delivery channel on an elastomeric substrate. Reproduced with permission from ref. ^23^. Copyright 2015, American Association for the Advancement of Science. **e** Syringe-injectable mesh electronics, illustrating a minimally invasive strategy for deploying sensor and interconnect networks into soft biological environments. Reproduced with permission from ref. ^24^. Copyright 2015, Macmillan Publishers Limited. **f** Wearable and disposable sweat-based glucose-monitoring system with a multistage transdermal drug-delivery module based on thermally actuated microneedles. Reproduced with permission from ref. ^49^. Copyright 2017, American Association for the Advancement of Science.

The tissue interface is not a passive boundary; it determines whether a drug channel remains patent, a sensor remains calibrated and an implant can operate chronically. Most biological tissues are soft, hydrated and viscoelastic, with moduli commonly in the kPa to low-MPa range (Fig. 2a, b). Conventional electronic materials, including silicon, metals and rigid printed circuit boards, are orders of magnitude stiffer. This mismatch results in interfacial shear, micromotion injury and chronic immune activation. The problem is especially severe in the brain and heart, where small deformations occur continuously during respiration, pulsation and movement^19–22^.

Insights from soft neural bioelectronics provide useful design principles for drug-delivery systems that must operate at mechanically delicate and chronically active tissue interfaces. Minev et al. developed electronic dura mater, or e-dura, from a soft silicone substrate, stretchable gold interconnects, and compliant electrodes^23^. The device mimicked the mechanics of the dura and withstood repeated deformation at the spinal-cord interface (Fig. 2c, d). Although its therapeutic outputs were mainly electrical and chemical stimulation, the principle is transferable: drug-delivery devices that contact neural tissue should deform with the host rather than impose a rigid boundary. This strategy supports long-term multimodal compatibility with soft neural tissue, but it also increases system complexity because soft interconnects, encapsulation and external connections remain difficult to be integrated with drug reservoirs and wireless modules. Liu and colleagues demonstrated a complementary strategy using syringe-injectable mesh electronics^24^. Their ultraflexible mesh could be loaded into a syringe, injected into tissue, and unfolded into a distributed network (Fig. 2e). A therapeutic system does not have to enter the body as a monolithic implant; it can be delivered through a needle or catheter and then deploy into a larger functional interface. The approach reduces the surgical footprint, but injectable electronics complicate positioning, fluidic integration, and retrieval. For wireless drug delivery, they also raise significant issues about reservoir, valve, and power-coil placement.

Hydrogels, elastomers and ultrathin polymers are promising material candidates for soft biointerfaces. Hydrogels are attractive because their high water content and tunable mechanics can mimic the extracellular matrix. They can also be engineered to be ionic, conductive, adhesive or drug loaded. Elastomers such as polydimethylsiloxane (PDMS) and polyurethane provide robust stretchability and are widely used for soft packaging, microneedle backings, and implantable reservoirs (Fig. 2f). Ultrathin polyimide and parylene devices remain stiff in bulk, but their bending stiffness decreases sharply with thickness, enabling conformal mechanics. A recurring trade-off is that the softest materials often have poor handling, barrier performance, or microfabrication compatibility. Hydrogels can dry, swell, or delaminate, whereas ultrathin films can fold or tear during implantation. Ideal systems therefore often adopt hybrid mechanics, pairing a soft tissue-contacting layer with a thin flexible electronic layer and protected rigid islands for high-performance systems^25–27^.

## Bioadhesion and immune evasion

**Fig. 3:**
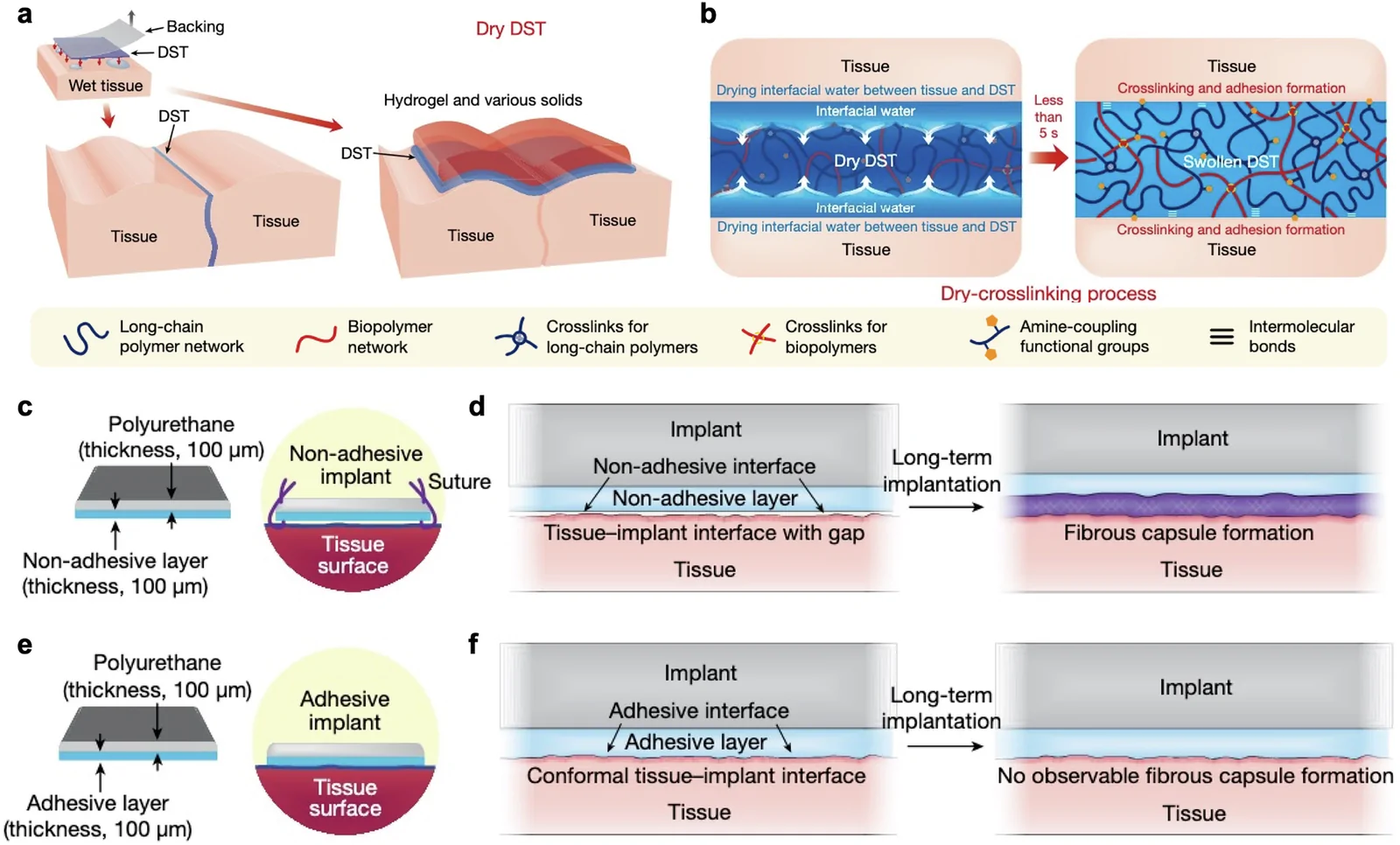
Soft adhesive and anti-fibrotic interfaces for stable tissue-device integration. **a**, **b** Dry double-sided tissue tape (DST) adheres to wet tissues by first removing interfacial water and then forming physical and covalent crosslinks with tissue surfaces. This dry-crosslinking mechanism enables rapid adhesion of hydrogels and other solids to wet biological substrates. Reproduced with permission from ref. ^28^. Copyright 2019, Springer Nature Limited. **c**, **d** A non-adhesive implant forms an interfacial gap after placement and can develop a fibrous capsule during long-term implantation. **e**, **f** An adhesive implant forms a conformal tissue-implant interface through an adhesive layer and can mitigate observable fibrous capsule formation. Reproduced with permission from ref. ^29^. Copyright 2024, Springer Nature Limited.

Softness alone is insufficient when a device can slip, trap fluid or become encapsulated by collagen. Drug-delivery systems are especially vulnerable because fibrous capsules and protein fouling can obstruct microneedles, pumps, valves and diffusion pathways. Wet bioadhesion therefore becomes a functional requirement. Yuk and colleagues introduced a dry double-sided tape for rapid adhesion of wet tissues and devices^28^. The adhesive combined tissue-interpenetrating surface chemistry with a dissipative matrix that provided mechanical toughness (Fig. 3a, b). Although the study was not designed for drug delivery, it addressed a general interface problem, i.e., soft biomedical devices must attach to slippery, dynamic tissues without sutures. Its value lies in its ability to bond quickly and robustly in wet biological conditions, whereas its translational constraints include atraumatic removal, sterilisation, degradation and organ-specific safety.

Recently, an adhesive anti-fibrotic interface was reported by Wu and colleagues^29^. The adhesive implant-tissue interface was fabricated from interpenetrating polymer networks. Adhesive integration reduced inflammatory-cell infiltration and prevented observable fibrous capsule formation on multiple organs over weeks in animal models. Mechanistically, eliminating the gap between implant and tissue may reduce the niche for protein adsorption, immune-cell accumulation, and collagen deposition (Fig. 3c–f). For wireless drug delivery, such an interface is particularly significant because both biosensing and drug release depend on stable, intimate, and low-impedance coupling with the target tissue. Robust adhesion can mechanically stabilize the device while simultaneously reshaping the local immune microenvironment, thereby preserving sensor fidelity and release-channel patency during operation. Nevertheless, the long-term performance of adhesive biointerfaces remains context-dependent. Diseased, fibrotic, infected, or highly motile tissues may alter adhesion strength, interfacial transport, and foreign-body responses, underscoring the need for specific and long-term validation under clinically relevant conditions.

Surface chemistry provides a complementary strategy to immune evasion. Polyethylene glycol (PEG), zwitterionic polymers, phosphorylcholine-like coatings and hydrophilic brushes can reduce nonspecific protein adsorption. Anti-inflammatory coatings, including dexamethasone-eluting layers, can suppress acute inflammation. However, passive antifouling coatings often degrade over time, and drug-eluting coatings ultimately exhaust their payload. This limitation motivates the development of next-generation drug delivery systems with multilayer biointerfaces that integrate mechanical compliance, wet adhesion, and biochemical stealth^30–32^. Viewed through the framework of controllability and reversibility, biointerfaces are not merely determinants of biocompatibility or user comfort; they are integral components of the therapeutic control loop. They govern whether a programmed dose reaches the target tissue with the intended timing and whether sensing signals remain sufficiently accurate to justify subsequent release decisions. Mechanical drift, interfacial fluid accumulation, fibrotic encapsulation, and coating degradation can progressively decouple electronic actuation from pharmacological outcomes by modifying local transport, sensor calibration, and pharmacokinetic/pharmacodynamic (PK/PD) latency. Accordingly, biointerface materials should be co-designed with reservoir architectures and release mechanisms, rather than considered as an isolated materials optimization problem.

## Wireless operation and actuation mechanisms

### Wireless power and communication

Wireless operation is central to translating electronically controlled drug delivery from laboratory instrumentation to clinical therapeutics. Tethered wires deliver power efficiently, but they compromise infection control, mobility, and patient acceptance. Wireless systems decouple the wearable or implantable therapeutic interface from the controller, battery, smartphone, or external transmitter. Common modalities include near-field magnetic coupling, near-field communication (NFC), Bluetooth, ultrasound, and magnetically triggered mechanical actuation^33–35^.

**Fig. 4:**
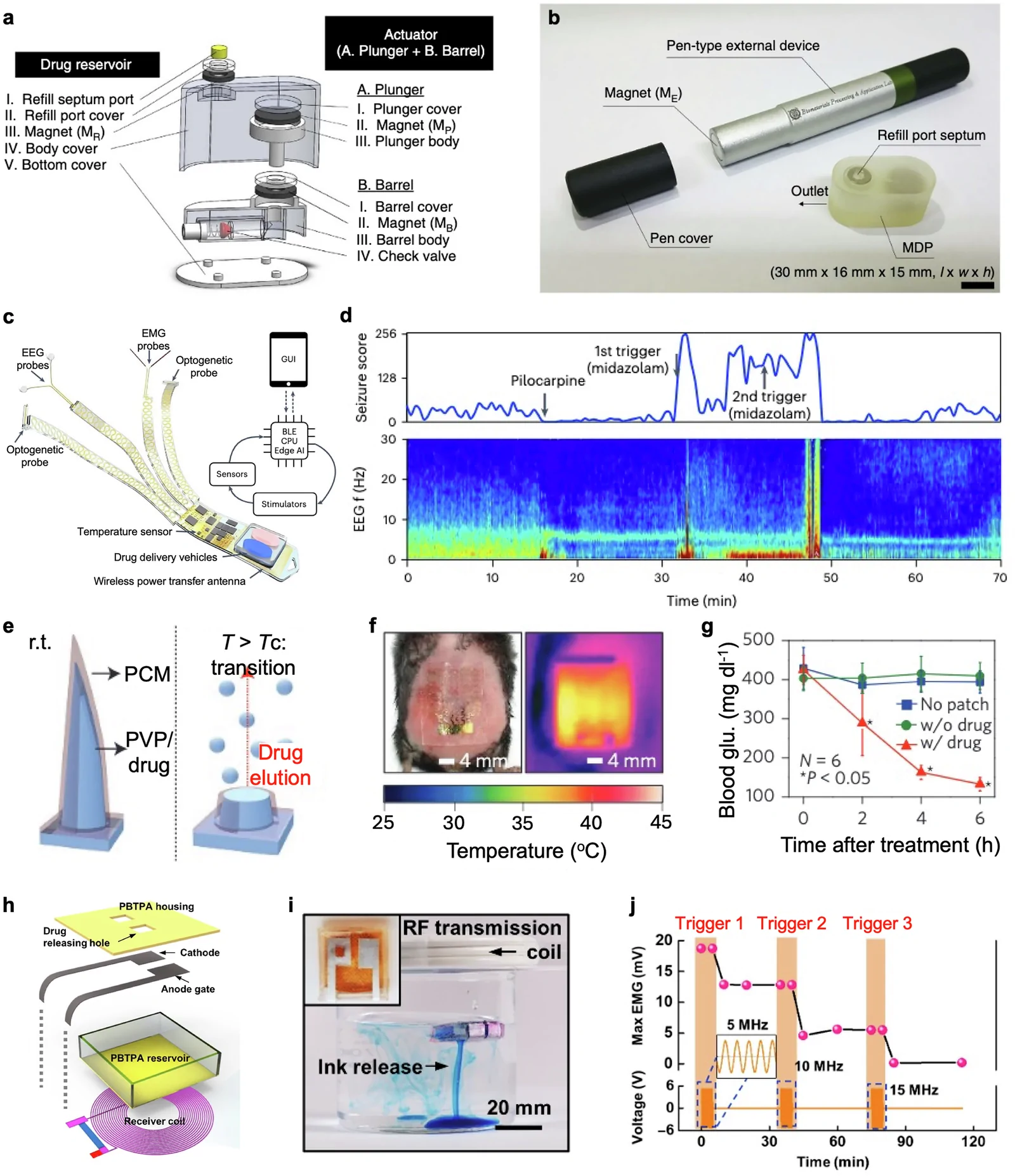
Wireless powering and triggered release mechanisms for on-demand drug delivery. **a** Exploded schematic of a magnetically actuated implantable and batteryless insulin pump. Reproduced with permission from ref. ^36^. Copyright 2017, Springer Nature Limited. **b** Photograph of the pen-type external actuator and implanted drug reservoir module. Reproduced with permission from ref. ^36^. Copyright 2017, Springer Nature Limited. **c** Architecture of a wireless, battery-free implant for multimodal closed-loop neuromodulation. Reproduced with permission from ref. ^37^. Copyright 2023, Springer Nature Limited. **d** Seizure-score and EEG traces showing feedback-triggered midazolam release. Reproduced with permission from ref. ^37^. Copyright 2023, Springer Nature Limited. **e** Thermoresponsive microneedle design showing drug elution above the phase-transition temperature. Reproduced with permission from ref. ^48^. Copyright 2016, Springer Nature Limited. **f** Skin photograph and thermal image illustrating local patch heating. Reproduced with permission from ref. ^48^. Copyright 2016, Springer Nature Limited. **g** Blood-glucose profiles after treatment with drug-loaded or control patches. Reproduced with permission from ref. ^48^., Copyright 2016, Springer Nature Limited. **h** Schematic of an electrochemically triggered bioresorbable valve and reservoir architecture. Reproduced with permission from ref. ^52^., Copyright 2020, American Association for the Advancement of Science. **i** RF-controlled release experiment showing wireless activation and model-payload ejection. Reproduced with permission from ref. ^41^. Copyright 2024, Springer Nature Limited. **j** Triggered-release response showing sequential activation events and electromyography output. Reproduced with permission from ref. ^41^. Copyright 2024, Springer Nature Limited.

Near-field magnetic coupling is widely used in subcutaneous and small-animal implants because it can deliver milliwatt-scale power over short distances while supporting bidirectional communication. Lee et al. reported an implantable battery-free device for on-demand, pulsatile insulin administration^36^. In this system, external magnetic actuation drove insulin injection without requiring an implanted battery (Fig. 4a, b). This design is attractive because the energy-storage unit and user interface remain outside the body, reducing the implanted components to a mechanical or microfluidic actuator. Such simplification lowers the electronic burden of the implant, but it also requires accurate patient alignment with the external source and places stringent demands on reservoir capacity and mechanical reliability.

More complex wireless systems that integrate sensing, data transfer and actuation have recently been developed. Ouyang et al. reported a wireless, battery-free implant for multimodal closed-loop neuromodulation in small animals^37^. The platform recorded physiological signals and triggered therapeutic intervention in freely moving animals (Fig. 4c, d). This architecture matters for drug delivery because sensing and actuation can be coordinated in a battery-free implant. The main limitation is the power budget, as recording, data processing, wireless communication, and release actuation all compete for energy. In humans, deeper implantation and variable alignment will further reduce efficiency. External transmitters, coils or ultrasound sources must therefore be designed around clinical geometry, not only bench-top coupling efficiency^38–40^.

NFC is particularly attractive for wearable drug-delivery patches because it enables short-range wireless power transfer and data exchange using low-cost readers. Wang et al. used an NFC module to control a spatiotemporally programmable, on-demand microneedle patch^41^. By contrast, Bluetooth is better suited to longer-range communication, smartphone-based programmability, and continuous data logging, although it typically requires an onboard battery or a larger harvested-power budget than passive NFC. Ultrasound provides deeper tissue penetration and can be spatially focused, making it promising for implants located beyond the practical range of inductive coupling. However, ultrasonic power transfer introduces safety constraints associated with tissue heating, cavitation, and tissue-specific acoustic absorption. Thus, no wireless modality is universally optimal. Wearable patches generally favour NFC or Bluetooth; shallow subcutaneous implants often benefit from inductive links; and deep miniaturized implants may require ultrasound, magnetoelectric transducers or hybrid power-management architectures^42–46^. Wireless links are therefore not merely convenient replacements for leads. They define the temporal resolution, power ceiling and safety envelope within which a release module can operate. A wireless command may be turned off immediately, but the drug bolus that it initiates cannot be withdrawn; consequently, wireless modality, actuation mechanism and reservoir architecture should be matched to the acceptable dosing latency and reversibility of the target indication.

## Triggered drug release mechanisms

At the core of wireless drug delivery is a transduction problem: how can an electromagnetic, acoustic, optical or digital command be converted into a defined chemical dose? Current strategies can be grouped into thermal or photothermal release, electrochemical gate opening, microfluidic or electroosmotic pumping, and acoustic or magnetic actuation^5,47^. These mechanisms should not be compared only by whether they release a drug rapidly. For therapeutic design, the more systematic comparison should include payload capacity, dose accuracy, release kinetics, reversibility or refillability, wireless power budget, drug stability, and the latency between sensed physiology and pharmacological effect (Table 1).

**Table 1:** Representative design constraints for wireless soft-bioelectronic drug-delivery systems

Representative system	Configuration	Wireless/actuation mode	Payload or dosing feature	Controllability and reversibility	Translational constraint highlighted
Magnetically actuated insulin pump^36^	Implantable pump	External magnetic actuation; battery-free	Insulin dose controlled by the number of magnetic applications	Pulsatile and user-triggered; refillable reservoir design	Dose depends on mechanical actuation reliability and reservoir access
Spatiotemporal on-demand microneedle patch^41^	Wearable patch	Wireless-powered digital control with electrically triggered membranes	Drug release to targeted sites smaller than 1 mm^2^ with response below 30 s	Spatially addressable and rapid, but release remains chemically irreversible	Requires coupling of digital control to drug stability and skin transport
Bioresorbable electrochemical-valve device^52^	Implantable reservoir array	Wireless power-harvesting unit triggers electrochemical corrosion	Defined reservoirs release drugs after valve opening	Programmable multi-reservoir release; each opened gate is effectively one-use	Useful for staged therapy, but dosing is constrained by reservoir number and gate reliability
Closed-loop microneedle diabetes patch^57^	Wearable microneedle system	Printed electronics control sensor and electroosmotic pump	Integrated 1.45 ml insulin reservoir reported for the patch housing	Pump can titrate delivery while reservoir and sensor remain stable	Long-term use depends on sensor drift, pump fouling and insulin stability
Wireless seizure-rescue implant^80^	Soft implantable reservoir	Wearable EEG monitoring triggers wireless voltage induction	Local emergency drug release for seizure intervention	Event-triggered release; rapid intervention prioritized over repeated titration	Clinical translation requires reliable detection and fail-safe triggering
Wireless neuropharmacology probe^87^	Implantable neural probe	Bidirectional wireless communication with miniaturized pump	Dose-dependent, repeatable drug responses; refillable reservoirs reported	Programmable local infusion with electrophysiology readout	Local PK, tissue injury and repeated refillability define chronic use

Thermoresponsive microneedles remain among the most compelling demonstrations of wearable closed-loop drug delivery. Lee et al. developed a graphene-based electrochemical patch that integrated thermoresponsive microneedles for diabetes monitoring and treatment^48^. In this platform, gold-doped graphene and a gold mesh enhanced sweat-glucose sensing, whereas integrated temperature, humidity, and pH sensors corrected for confounding sweat conditions. The therapeutic module consisted of metformin-loaded polymeric microneedles coupled to phase-change materials; local heating melted the phase-change layer and triggered transcutaneous drug release (Fig. 4e–g). The major strength of this system lies in its elegant integration of sensing and therapy within a flexible patch. However, sweat glucose remains an indirect and physiologically variable surrogate for blood glucose, and thermal actuation must be tightly regulated to avoid skin irritation. A subsequent wearable, disposable sweat-based system advanced this point-of-care concept by enabling multistage release^49^. In that design, multiple phase-change nanoparticles were embedded within hyaluronic acid microneedles, improving programmable dosing. Nevertheless, the system still depended on sweat sampling and heater-mediated release, both of which are sensitive to interindividual physiology and environmental conditions^50,51^.

Electrochemical gates provide more discrete and spatially precise actuation. Koo et al. reported a wirelessly controlled, bioresorbable device with active valves based on electrochemically triggered crevice corrosion^52^. Metallic gates sealed reservoirs and dissolved when triggered, releasing local anaesthetic. The key principle is that a small electrical stimulus initiates corrosion of a thin biocompatible metal, thereby converting an electronic command into a fluidic opening (Fig. 4h). The advantage is fast, robust, and binary release with bioresorbable components. The limitation is that many dissolvable gates are one-shot: once opened, the same reservoir cannot be resealed. Refillable architectures or multireservoir arrays can compensate, but they increase volume and complexity^53,54^. Wang et al. translated a related principle to a wearable microneedle format^41^. Their spatiotemporal on-demand patch used PLGA microneedles coated with an approximately 150 nm gold layer. A low-voltage trigger dissolved selected gold domains through crevice corrosion, exposing the drug-loaded microneedle core and initiating release. The system achieved submillimetre spatial control and responses within tens of seconds, while NFC enabled digital programming. This example directly links wireless communication, electronic switching and material-gated release. Its advantage is high spatial addressability and scalable microneedle fabrication. Its limitation is partial irreversibility, because each activated microneedle becomes an opened dose unit. The approach is therefore well suited to scheduled bolus dosing, but less suited to continuous titrated infusion unless many addressable elements are available^55,56^.

Microfluidic and electroosmotic pumps are better suited to rate-controlled infusion. Liu et al. reported a wearable microneedle patch for closed-loop diabetes management^57^. The system integrated a graphene-composite ink glucose sensor on hollow microneedles, a PEG-functionalised electroosmotic micropump, and a control circuit. When the interstitial glucose signal exceeded a threshold, the pump delivered insulin through hollow microneedle channels. Electroosmosis manipulates liquid through nanoporous membranes under an electric field. Its advantage is quantitative and repeatable flow rather than one-shot gate opening. The PEG-based antifouling strategy extended pump stability from days to weeks. The disadvantages are power consumption, membrane fouling, finite reservoir volume, and incomplete wireless integration for practical human use^58,59^.

Acoustically mediated actuation provides a distinct strategy for wireless drug delivery by converting remotely modulated sound fields into localized mechanical forces that promote transdermal transport. Xu et al. reported an acoustic metamaterial-driven platform for rapid, on-demand transdermal drug delivery in acute disease management^60^. In this system, engineered acoustic structures concentrated mechanical energy at the skin interface, thereby enhancing delivery without implanted electronics. This class of device is attractive for acute or intermittent therapy, but its translational value still depends on dose precision, tissue-specific coupling, and how rapidly the released payload reaches the pharmacological target^61–66^.

## Integrated closed-loop systems and clinical applications

**Fig. 5:**
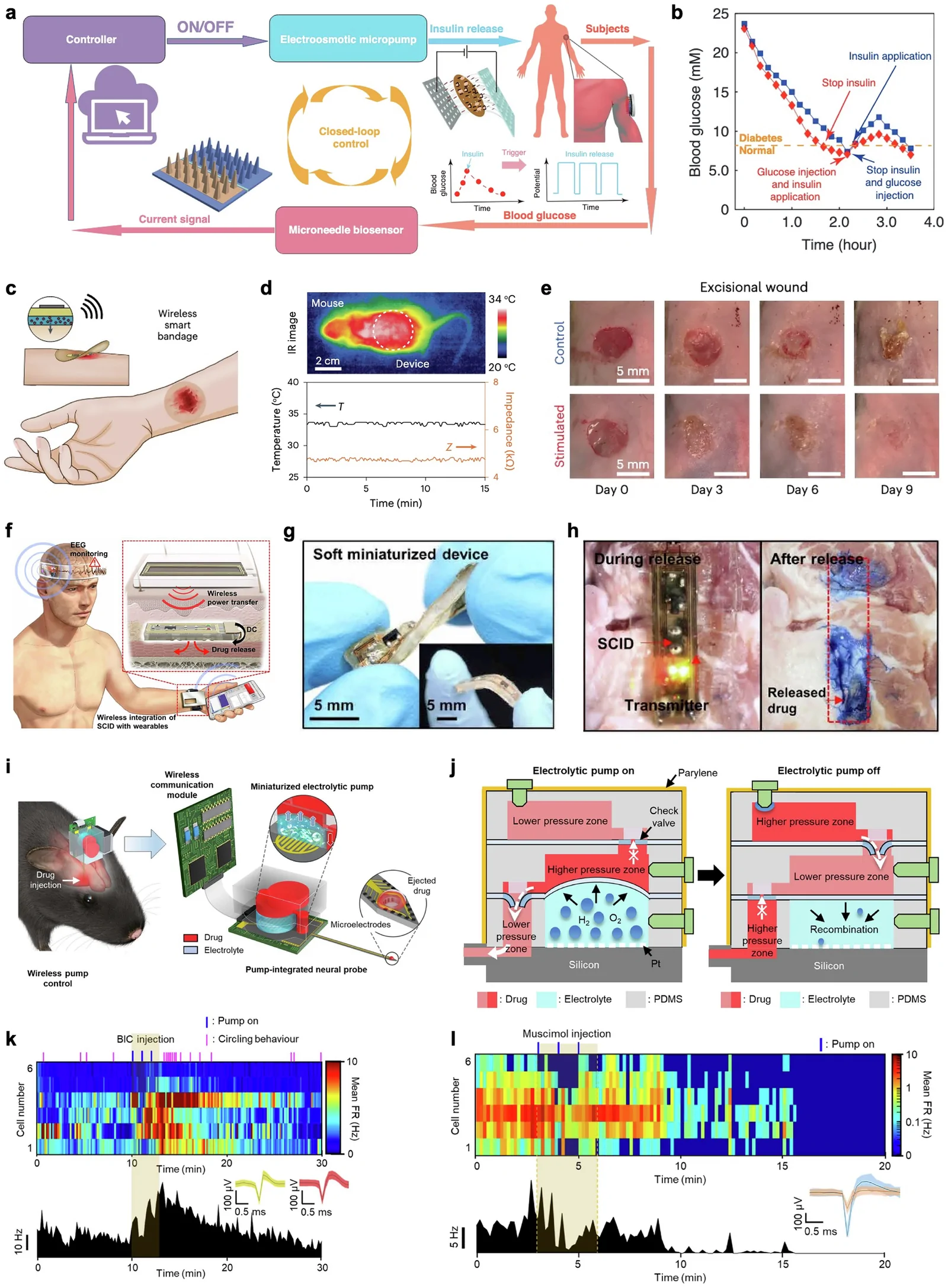
Closed-loop and application-oriented soft wireless drug-delivery systems. **a** Schematic of a wearable closed-loop microneedle system coupling glucose sensing, electroosmotic pumping and insulin release. Reproduced with permission from ref. ^57^. Copyright 2024, Springer Nature Limited. **b** Blood-glucose profile illustrating closed-loop insulin administration. Reproduced with permission from ref. ^57^. Copyright 2024, Springer Nature Limited. **c** Schematic of a wireless smart bandage for wound monitoring and therapy. Reproduced with permission from ref. ^70^. Copyright 2023, Springer Nature Limited. **d** Infrared image and temperature/impedance traces during device operation on a mouse wound. Reproduced with permission from ref. ^70^. Copyright 2023, Springer Nature Limited. **e** Representative wound images comparing control and stimulated groups over time. Reproduced with permission from ref. ^70^. Copyright 2023, Springer Nature Limited. **f** Wireless integration of a soft implantable drug-delivery device with wearable monitoring. Reproduced with permission from ref. ^80^., Copyright 2021, American Association for the Advancement of Science. **g** Photograph of the soft miniaturized implantable device. Reproduced with permission from ref. ^80^., Copyright 2021, American Association for the Advancement of Science. **h** In vivo images showing drug release during and after device activation. Reproduced with permission from ref. ^80^. Copyright 2021, American Association for the Advancement of Science. **i** Wireless neural-probe system integrating a communication module, miniaturized electrolytic pump and pump-integrated neural probe. Reproduced with permission from ref. ^87^. Copyright 2022, Springer Nature Limited. **j** Electrolytic-pump operating mechanism during pump-on and pump-off states. Reproduced with permission from ref. ^87^. Copyright 2022, Springer Nature Limited. **k** Neural firing-rate heat map and behavioral response after BIC injection. Reproduced with permission from ref. ^87^. Copyright 2022, Springer Nature Limited. **l** Neural firing-rate heat map after muscimol injection during wireless pump activation. Reproduced with permission from ref. ^87^. Copyright 2022, Springer Nature Limited.

Closed-loop drug delivery is most compelling when therapeutic output must be matched to an evolving biological state. Figure 5 highlights representative soft wireless systems that couple physiological monitoring with programmable actuation and local therapy. The key design issue is that chemical dosing carries memory: after release, the payload persists until it diffuses, binds, degrades, or clears. Closed-loop drug delivery therefore requires conservative feedback thresholds, reliable sensors and release modules whose kinetics are matched to the disease timescale. Examples that do not directly release drugs are included in this section only when they provide transferable architectures for soft tissue coupling, wireless power, closed-loop sensing or implantable control. Across therapeutic applications, the critical question is not simply whether a system is closed loop, but what therapeutic modality the feedback loop controls. Unlike electrical stimulation, which can be switched on or off within milliseconds, drug-based interventions are intrinsically limited by pharmacokinetic and pharmacodynamic (PK/PD) processes, including release kinetics, tissue transport, receptor binding, and systemic clearance. These temporal constraints make PK/PD latency a defining design parameter, guiding the selection of delivery architectures ranging from one-shot gates and multireservoir arrays to titratable pumps and refillable implants.

## Wearable therapeutics for metabolic and skin diseases

Diabetes remains the most intuitive testbed for closed-loop soft drug delivery because both the biomarker and therapeutic target are well defined. The wearable microneedle patch reported by Liu et al. illustrates how this logic can be translated into a skin-interfaced device (Fig. 5a, b)^57^. The system integrates a graphene-Prussian blue ink glucose sensor on hollow microneedles, a PEG-functionalised electroosmotic micropump, and a control circuit. Interstitial glucose is measured through the microneedle interface, and insulin is delivered through the same hollow channels when glucose exceeds a threshold. Compared with sweat-based glucose patches, this design is closer to clinical continuous glucose monitoring because it samples interstitial fluid and delivers insulin rather than an indirect antihyperglycaemic agent. Its main advance is not only the use of microneedles, but also the co-design of sensing chemistry, antifouling pumping and dose actuation in a compact patch.

This work also underscores the engineering gap between a compelling closed-loop prototype and a practical wireless therapeutic system. The reported patch achieved autonomous glucose regulation in diabetic rats, including corrective feedback after a glucose challenge. However, its control strategy remained threshold based, its reservoir capacity was finite, and its electronic components still require further miniaturization and wireless integration for routine use. For human diabetes management, a soft closed-loop patch would require predictive control algorithms, robust sensor calibration, fail-safe insulin-delivery limits and a manufacturable disposable interface. This platform should therefore be viewed as an important advance in closed-loop patch engineering, rather than as a near-term replacement for mature artificial-pancreas systems^67–69^.

Wound care provides a different entry point because the target tissue is accessible, visually inspectable and highly dynamic. The wireless smart bandage developed by Jiang and colleagues shows how a soft bioelectronic dressing can monitor wound state and actuate therapy in response (Fig. 5c–e)^70^. The platform combines wirelessly powered sensing and stimulation circuits with conductive hydrogel electrodes that support tissue contact and on-demand detachment. In preclinical wound models, impedance and temperature measurements were used to follow wound physiology, and electrical stimulation was delivered to accelerate repair. The reported improvement in healing rate and dermal remodelling highlights the value of closing the loop at the wound surface^71^. Although this smart bandage uses electrical stimulation rather than drug release, it is included here because it illustrates a soft bioelectronic architecture that can readily be extended to programmable drug delivery. Chronic wound therapy should adapt to dynamic changes in infection, exudate, inflammation and tissue regeneration, enabling antimicrobial, anti-inflammatory or pro-regenerative interventions to be delivered only when needed. A future drug-delivering dressing could therefore retain the same sensing, wireless communication and feedback-control framework while replacing or augmenting electrical stimulation with reservoir-based drug release. More broadly, stretchable dressings, exudate-responsive systems, battery-free smart wound dressings, wireless needle-array bandages, multifunctional hydrogel dressings and flexible wound electronics could illustrate a converging design paradigm in which sensing, actuation and on-demand therapy are integrated into adaptive wound care platforms^72–79^.

## Minimally invasive implants and wireless neurological and cardiovascular care

Neurological disease illustrates the potential advantage of wireless pharmacology over conventional systemic dosing. Seizures, pain and circuit dysfunction often require rapid, localized intervention, whereas systemic drugs can produce dose-limiting off-target effects. Joo et al. addressed this challenge using a soft implantable drug-delivery device wirelessly integrated with wearable electronics for seizure rescue (Fig. 5f–h)^80^. In this system, EEG sensing, power transmission, and control were externalized into wearable modules, allowing the implanted component to remain soft and miniaturized. When seizure-like activity was detected, wireless power triggered electrolysis within the reservoir, releasing diazepam from the soft implant^81–84^. This architecture is important because it decouples the therapeutic interface from the computational and power burdens that often make implants rigid, bulky or surgically intrusive. It also demonstrates a complete rescue loop comprising physiological monitoring, wireless decision transfer, wireless actuation and local pharmacological intervention. At the same time, it defines the safety requirements that any autonomous drug-delivery implant must satisfy. Such systems must distinguish true pathological events from false positives, avoid missed events, track residual drug volume and fail safely when reservoirs are depleted, or wireless coupling is insufficient. Because the actuator releases a finite drug payload, chronic use will also require credible strategies for reservoir refilling, device replacement or programmed degradation^85,86^.

Yoon et al. broadened the neurological application space from emergency intervention to wireless neuropharmacology (Fig. 5i)^87^. The as-fabricated platform integrates a neural probe, a miniaturized electrolytic pump, and bidirectional wireless communication into a head-mounted system for freely behaving mice. The pump enables dose-controlled local drug infusion, while the electrode array records neural activity during and after pharmacological perturbation. By combining drug delivery, electrophysiology and behavioral analysis in the same animal, including during social-interaction experiments, this system provides a powerful architecture for causal interrogation of neural circuits. From a translational perspective, its integration of dose-controlled infusion, refillable reservoirs, simultaneous neural recording and wireless control enables mechanistic studies that are difficult to achieve with conventional pharmacology. Yet clinical deployment would require substantial redesign: the small-animal head-mounted format would need to be miniaturized or anatomically adapted, invasive brain implantation would need to be justified by a clear therapeutic indication, reservoir capacity would need to support chronic use, and both fluidic and electrical interfaces would need to remain stable over long timescales. Nevertheless, the study points to an important design principle for future implantable drug-delivery systems: the most informative platforms will not merely release drugs on command, but will also measure the physiological consequences of release and use that information to refine subsequent dosing.

Translational readiness depends less on the sophistication of any individual component than on whether the overall device architecture is matched to the intended disease context. Wearable patches and dressings can be removed, replaced and visually inspected, making them well suited to accessible applications such as wound care and metabolic monitoring. However, their performance remains constrained by limited payload capacity, variable adhesion, motion artefacts and user-dependent sampling. By contrast, implanted neurological and cardiovascular systems enable localized drug delivery to otherwise inaccessible targets but require stronger evidence for long-term biocompatibility, refillability, fail-safe operation and reliable wireless performance. These contrasting requirements argue against a universal ranking of device formats and instead support a failure-mode-driven framework in which architecture is selected according to the dominant translational constraint. Beyond the representative examples highlighted in Fig. 5, the broader landscape includes cardiac tissue–electronics hybrids, wearable systems for movement disorders, smart contact lenses, intraoral patches, ingestible electronics, mucosal interfaces, microrobotic delivery platforms, personalized 3D-printed formulations, enzymatic biofuel-cell-powered release systems and fully integrated ingestible electronic platforms^88–102^. Together, these advances illustrate the expanding range of soft bioelectronic architectures that can be tailored to different therapeutic settings while following the same principles of context-specific design.

## Challenges and future perspectives

Soft wireless drug-delivery systems have advanced from isolated materials, biosensors and actuation demonstrations towards integrated therapeutic platforms. Their translational potential will depend on whether these components operate as coordinated dosing systems under realistic physiological, manufacturing and regulatory constraints. The first challenge is payload design. Reservoir capacity, drug concentration, stability during storage, leakage resistance and release kinetics must be matched to the intended therapeutic window. A high-resolution actuator has limited value if it cannot maintain drug potency or deliver a clinically meaningful dose over the required duration.

The second challenge is long-term stability across both the biointerface and the drug path. Soft materials are mechanically attractive but chemically vulnerable. Hydrogels can dehydrate or swell, elastomers can absorb small molecules, thin metal traces can fatigue, and biofluids can penetrate encapsulation. Sensors introduce additional drift because biorecognition layers foul or lose activity. Closed-loop systems are only as safe as their sensing and dosing modules. A glucose patch that overestimates glucose can overdose insulin, whereas a seizure-rescue implant that releases too late may miss the therapeutic window.

The third challenge is biocompatibility beyond acute toxicity. Foreign-body response, fibrosis and bacterial biofilms can degrade performance even when materials are cytocompatible in vitro. Adhesive anti-fibrotic interfaces are promising, but they must be tested under clinically realistic conditions, including diabetic skin, infected wounds, arrhythmic myocardium and scarred neural tissue. Drug release itself can alter the interface. Antibiotics reshape local microbiomes, steroids suppress healing, and neuroactive drugs may change circuit excitability. These coupled material and pharmacological effects should be considered together.

The fourth challenge is feedback governance. Artificial intelligence (AI) and model-predictive control could personalize therapy by forecasting glucose trajectories, seizure onset, wound infection risk or rehabilitation state and adjusting treatment accordingly. However, adaptive algorithms present a regulatory challenge because device behavior may evolve after deployment. Regulators are therefore likely to require bounded learning, explainable decision-making, robust cybersecurity, and clearly defined fail-safe states. These requirements are particularly stringent for drug-delivery systems, where an erroneous control decision may result in irreversible chemical exposure rather than a reversible stimulation event. Emerging work on AI-guided drug-delivery design, AI-based medication assessment and autonomous closed-loop body-integrated devices illustrates both the potential of adaptive therapeutic control and the substantial governance framework required for its safe clinical translation^103–105^.

A practical translational roadmap should begin from the dominant clinical failure mode. Interface failure calls for improved adhesion, compliance and immune compatibility. Dosing failure calls for better reservoir design, drug stability, release kinetics and refillability. Control failure calls for robust sensing, conservative thresholds and fail-safe algorithms. Deployment failure calls for wireless power, manufacturability, replacement strategy and regulatory design matched to the intended use context. Near-term translation will probably occur in replaceable wearable systems for diabetes, wound care and pain, where failure is visible, and devices can be removed. Subsequent platforms may include subcutaneous or catheter-delivered implants for seizure rescue, local analgesia and postoperative inflammation, where localized therapy may justify greater invasiveness.

In summary, soft bioelectronics for wireless drug delivery is maturing from device demonstrations into therapeutic systems. The studies discussed here show rapid progress in soft interfaces, wireless power transfer, electrochemical gates, microneedles, micropumps and closed-loop control. They also show that no single design resolves all translational barriers. A useful next step is to select components around the therapeutic failure mode and the controllability of chemical dosing. Future platforms must be soft enough to coexist with living tissue, wireless enough to remain minimally invasive, intelligent enough to personalize therapy, and pharmacologically constrained enough to deliver the right dose at the right time.

## Data Availability

No datasets were generated or analysed during the current study.
